# Lung disease burden assessment by oscillometry in a systematically disadvantaged urban population experiencing homelessness or at-risk for homelessness in Ottawa, Canada from a prospective observational study

**DOI:** 10.1186/s12890-022-02030-x

**Published:** 2022-06-16

**Authors:** Smita Pakhale, Carly Visentin, Saania Tariq, Tina Kaur, Kelly Florence, Ted Bignell, Sadia Jama, Nina Huynh, Robert Boyd, Joanne Haddad, Gonzalo G. Alvarez

**Affiliations:** 1 Department of Medicine, The Ottawa Hospital, 501 Smyth Road, Ottawa, ON K1H 8L6 Canada; 2 The Ottawa Hospital Research Institute (OHRI), Ottawa, Canada; 3 University of Ottawa, Ottawa, Canada; 4 University of Toronto, Toronto, Canada; 5 Community (Peer) Researcher, The Bridge Engagement Centre, Ottawa, Canada; 6 Oasis, Sandy Hill Community Health Centre, Ottawa, Canada; 7 Canadian Mental Health Association, The Ottawa Branch, Ottawa, Canada

**Keywords:** Community-based research, Patient engagement, Lung function, Asthma, COPD, Homelessness

## Abstract

**Rationale:**

Oscillometry is an emerging technique that offers some advantages over spirometry as it does not require forced exhalation and may detect early changes in respiratory pathology. Obstructive lung disease disproportionately impacts people experiencing homelessness with a high symptoms burden, yet oscillometry is not studied in this population.

**Objectives:**

To assess lung disease and symptom burden using oscillometry in people experiencing homelessness or at-risk of homelessness using a community-based participatory action research approach (The Bridge Model™).

**Methods:**

Of 80 recruited, 55 completed baseline oscillometry, 64 completed spirometry, and all completed patient-reported outcomes with demographics, health, and respiratory symptom related questionnaires in the Participatory Research in Ottawa: Management and Point-of-Care for Tobacco Dependence project. Using a two-tail t-test, we compared mean oscillometry values for airway resistance (R_5–20_), reactance area under the curve (A_x_) and reactance at 5 Hz (*X*_5_) amongst individuals with fixed-ratio method (FEV_1_/FVC ratio < 0.70) and LLN (FEV_1_/FVC ratio ≤ LLN) spirometry diagnosed chronic obstructive pulmonary disease (COPD). We compared mean oscillometry parameters based on participants’ COPD assessment test (CAT) scores using ANOVA test.

**Results:**

There was no significant difference between the pre- and post- bronchodilator values of R_5–20_ and A_x_ for the fixed ratio method (*p* = 0.63 and 0.43) and the LLN method (*p* = 0.45 and 0.36). There was a significant difference in all three of the oscillometry parameters, R_5–20_, A_x_ and X_5_, based on CAT score (*p* = 0.009, 0.007 and 0.05, respectively). There was a significant difference in R_5–20_ and A_x_ based on the presence of phlegm (*p* = 0.03 and 0.02, respectively) and the presence of wheeze (*p* = 0.05 and 0.01, respectively). Oscillometry data did not correlate with spirometry data, but it was associated with CAT scores and correlated with the presence of self-reported symptoms of phlegm and wheeze in this population.

**Conclusions:**

Oscillometry is associated with respiratory symptom burden and highlights the need for future studies to generate more robust data regarding the use of oscillometry in systematically disadvantaged populations where disease burden is disproportionately higher than the general population.

*Trial* *Registration*: *ClinicalTrails.gov*—NCT03626064, Retrospective registered: August 2018, https://clinicaltrials.gov/ct2/show/NCT03626064

## Introduction

Spirometry is the most used lung function test for diagnosis of lung diseases and monitoring lung health including for many obstructive lung diseases (OLD), however, it has several limitations [1]. It relies on the user to be able to follow commands and to generate a forced exhalation, making it challenging to use in certain populations (i.e., the frail elderly, people with poor lung function, and children) [2, 3]. Twenty percent of patients find spirometry manoeuvre to be unacceptable and 31% find guiding statements about prolonged expiration while performing the test (“To keep blowing even though you do not feel anything is coming out”) to be moderately or seriously difficult to follow [4]. It also requires a highly skilled health care provider to coach the patient during the procedure and to analyze the flow-volume loop and determine its appropriateness. Furthermore, changes in spirometry parameters are undetectable in the early stages of small airway disease and may only become abnormal once there is significant pathology or a high degree of symptom burden [5].

Oscillometry is a technique that was first described in 1956 [6] and has gained increased recognition in its clinical ability to assess respiratory function. The forced oscillation technique (FOT) uses forced oscillation sound waves superimposed on normal, spontaneous breathing to measure airway impedance, resistance, and reactance [7]. The oscillometer measures respiratory system impedance (a measure of the relationship between pressure and flow changes during normal tidal breathing) which has two components, resistance (*R*_*rs*_) and reactance (*X*_*rs*_). *R*_*rs*_ is a measure of airway calibre and *X*_*rs*_ is a measure of the viscoelastic properties and stiffness of the airways [8]. Unlike spirometry, oscillometry does not rely on forceful expiration, therefore, it requires less effort and co-operation, facilitating its use in diverse populations (i.e., pediatrics, elderly, and ventilated and sleeping patients) [9–12]. By virtue of being a simpler and non-effort dependent test, less patient and operator training may be required for oscillometry compared to spirometry. Oscillometry results are analyzed by a computer program that compares the patient’s results to normative data based on the patient’s demographic information, thereby individualizing results [13]. Oscillometry has been found to correlate with patient self-reported symptoms of chronic obstructive pulmonary disease (COPD) and asthma [9, 14, 15] and to be as sensitive as spirometry in assessing lung disease in epidemiological studies [16–19]. Despite its ability to detect small airway disease and disease burden, it is unclear whether oscillometry can distinguish between asthma and COPD diagnoses [20–22]. Oscillometry resistance and reactance values at low frequency have been shown to correlate strongly with transpulmonary resistance measured by esophageal manometry and other traditional small airway measures [23]. Clement et al. [24], demonstrated that oscillometry was a sensitive tool to differentiate between healthy patients and those with respiratory disease (even in the presence of a normal FEV_1_) and that the sensitivity to detect symptoms was similar between spirometry and oscillometry. Another study by Van Nord et al. [25], found that oscillometry parameters performed better than spirometry in differentiating amongst patients with asthma, chronic bronchitis, and emphysema. Despite the clinical practicality and sensitivity of oscillometry in detecting lung disease and symptom burden, oscillometry is limited by its inability to differentiate between obstructive and restrictive lung disease and intra-pulmonary versus extra-pulmonary central airway obstruction [16]. Overall, current literature supports oscillometry as a valuable addition to spirometry in evaluating airway disease, including its potential to detect early changes in small airway pathology [5]. Despite the potential widespread application of oscillometry, its use is limited by the paucity of data regarding reference values for oscillometry parameters. Those that do exist [25–29] are derived from small-scale studies consisting of mainly healthy Caucasians who do not smoke, without a personal history of lung disease [26, 27]. The use of oscillometry in people in urban settings who identify as experiencing homelessness or being at-risk for homelessness, is non-existent, despite a disproportionately higher prevalence of OLDs and associated symptom burden in this population related to disproportionately higher prevalence of addictions (including tobacco) as compared to the general population [28]. Therefore, there is a need for studies to assess the role of oscillometry in measuring lung function and disease burden in this systematically disadvantaged population. To our knowledge, this is the first study to assess lung disease and disease burden using oscillometry in a population that is underserved and experiencing homelessness or at-risk for homelessness.

In this study, we assessed obstructive lung disease (diagnosed by hand-held spirometry) and symptom burden using oscillometry in people who self-identify as experiencing homelessness or at-risk homelessness in downtown Ottawa. We compared the oscillometry values to spirometry values and to patients’ self-reported symptoms [28]. We hypothesized that oscillometry values would correlate with spirometry values and that oscillometry values would vary based on patients’ symptom burden of OLD, such as COPD.

## Methods

### Data and measures

Data were obtained from the baseline assessment of the Participatory Research in Ottawa: Management and Point-of-Care for Tobacco Dependence (PROMPT) project, a prospective observational cohort study using a community-based participatory action research (CBPAR) approach (The Bridge Model™) in partnership with people with lived experience similar to the project participants, including homelessness and tobacco smoking (ClinicalTrails.gov identifier NCT03626064, retrospectively registered: 10/08/2018). To be eligible, participants had been (1) currently living in Ottawa for at least 3 months, (2) been 16 years or older, (3) have been using street drugs (other than recreational marijuana or alcohol) in the past year, and (4) have smoked tobacco in the past 7 days. Full study details of CBPAR approach have been reported in previous PROMPT publications [28–30]. Briefly, all participants were provided a 6-month community-based intervention to reduce tobacco smoking and improve overall quality of life. During the 6-month study period, all participants had access to free nicotine replacement therapy, one-on-one nurse counselling twice per week, ongoing peer support, peer-led and co-created weekly life-skills workshops, and access to a safe non-judgemental, low-threshold community-based research space. All study-related activities, including intervention delivery and data collection (spirometry and oscillometry administration), were completed in a community setting by community peer researchers, people with lived experience of homelessness, at-risk for homelessness, and poverty.

At baseline, participants completed demographic and health-related questionnaires, including the BOLD core questionnaire used in the CanCOLD study which aims to evaluate respiratory symptoms (cough, phlegm, wheezing, shortness of breath) [31] and the COPD assessment test (CAT), an open-access disease specific questionnaire [32]. Participants who consented and were physically able underwent pre- and post-bronchodilator hand-held spirometry, oscillometry measurements (R_5–20_, a presumed measure of small airway resistance that increases in OLD; A_x_, a measure of the area under the reactance curve that increases in OLD; and X_5_, a measure of the elastic properties of the airway that worsens (becomes more negative) in OLD [33]) and an expired carbon monoxide test at baseline and at the final 6-month follow up. Spirometry data collection methods and analysis are reported in two previous publications [28, 34]. Individuals were labelled with COPD based on spirometry using both the fixed ratio method (a post-bronchodilator FEV_1_/FVC ratio < 0.70) and the LLN (a post-bronchodilator FEV_1_/FVC ratio ≤ LLN). Participants were categorized with Asthma if they showed significant reversibility, defined as pre-bronchodilation FEV1/FVC ratio < 0.70 or ≤ LLN with an improvement of ≥ 12% and 200 cc in FEV1 or FVC post-bronchodilation. We determined Lower Limit of Normal (LLN) using NHANES III spirometric reference equations for Caucasian populations [35] as there was no appropriate reference data for participants who self-identified as Indigenous and East Asian [36]. Due to limited access to equipment in the community, lung volumes or body plethysmography was not performed. Furthermore, we found many of the participants have not felt safe going to hospital or clinical settings due to stigma [28, 37] and as such we found it most appropriate to conduct hand-held spirometry in a community-based setting.

### Oscillometry

All oscillometry data were obtained using the tremoflo device (Thorasys Inc. Montreal, QC), an oscillometry technique that employs the FOT [7]. The system was calibrated once daily with the reference test load of 2cmH2O.s/L provided with the system [28]. All oscillometry data was collected prior to tests requiring deep breaths (i.e., spirometry) to minimize the effects of lung volume. Prior to starting the testing, patients were instructed on proper technique (taking relaxed and stable breaths while seated in an upright posture, supporting one’s cheeks, and forming a tight seal around the mouthpiece). The test was repeated to ensure a minimum of three, 30 s, technically acceptable measurements with a coefficient of variation of less than 15%. Volume, flow, and pressure tracers were visually inspected to identify the presence of artefact and tests with artefact were excluded. Due to the small sample size, there was no control group available to obtain normative values or calculate Z-scores. Furthermore, the reference values published in other studies cannot be compared to our study population as they tested primarily older subjects [27], utilized different devices [38, 39], or were not conducted in a North American population [26]. Oscillometry testing was optional for participants (i.e., they could opt out) as it was completed at the end of multiple questionnaires and procedures at a single visit. This decision was made in partnership with community peer researchers to reduce participant burden.

### Statistical analysis

Mean oscillometry values for airway resistance (R_5–20_), reactance area under the curve (A_x_) and reactance at 5 Hz (*X*_5_) were compared amongst individuals diagnosed with COPD based on spirometry using both the fixed ratio method (a post-bronchodilator FEV_1_/FVC ratio < 0.70) and the LLN (a post-bronchodilator FEV_1_/FVC ratio ≤ LLN) using a two-tailed t-test. Any individuals with missing data (i.e., oscillometry, spirometry, CAT score, symptoms questionnaire) were excluded from the analysis. Reasons for missing data are provided in the results section.

The mean oscillometry parameters were compared based on the participants’ CAT scores using the ANOVA test. In subgroup analyses, the mean oscillometry parameters were compared based on the presence of specific symptoms (cough, phlegm, wheeze, and shortness of breath), as well as, compared amongst subjects with a diagnosis of asthma versus COPD using two-tailed t-tests.

## Results

From March to August 2016, community peer researchers used a social network approach to recruit eighty participants from Ottawa’s urban population who identified as experiencing homelessness or at-risk for homelessness (< 5% potential participants contacted but not enrolled). Of the eighty participants, sixty-four completed spirometry and fifty-five completed both spirometry and oscillometry at baseline. Of the participants that did not undergo spirometry testing, five did not give a reason for refusing, two reported experiencing a heart attack in the last three months, three reported experiencing a detached retina or migraine recently, one reported a respiratory infection in the previous three weeks and two reported using salbutamol in the six hours prior to the test or being unable to physically perform the spirometry test. Additionally, three spirometry tests were removed due to poor-quality tests (over 40% decrease in post-bronchodilation performance of inconsistent FEV_1_ and FVC outputs), for a total of 64 spirometry tests completed. The spirometry analysis is presented in a previous publication [28]. Nine additional participants opted out of the (optional) oscillometry test and provided numerous reasons, including running late to soup kitchens/shelters, withdrawal symptoms, or running out of patience after a lengthy baseline survey and clinical measurements.

Participants were mostly Caucasian males with an average age of 44 years. Over one third reported not completing high school and 80% of the participants reported some level of food insecurity. The average monthly income was between $1000–$1999. At study baseline, all participants had active tobacco smoking history and most participants reported smoking less than 25 cigarettes daily. Only 13 participants reported a physician-diagnosed lung disease. However, all participants reported some respiratory symptoms (cough, phlegm, shortness of breath, wheezing) impacting their ability to function as indicated by the CanCOLD questionnaire and CAT score (Table 1).

**Table 1:** Demographics Characteristics of participants enrolled in the PROMPT study who completed spirometry testing (n = 64) and who also completed oscillometry testing (n = 55)

Characteristic	Participants with spirometry (n = 55)	Participants with spirometry and oscillometry (n = 64)
Sex (male)	69%	67%
Age (SD)	43.9 (11.0)	44.0 (11.5)
BMI (SD)	25.9 (6.8)	25.8 (6.9)
*Education*		
College or university completed	6%	7%
Some college or university	27%	31%
High school graduate/GED	28%	27%
Elementary/ grade school or some high school	36%	31%
None	1.5%	2%
NA	1.5%	2%
*Ethnicity*		
Caucasian	78%	73%
Aboriginal (Metis, Inuit, First Nation)	19%	24%
Other	3%	3%
*Monthly* *income*		
$200–2999	11%	5%
$1000–1999	43%	44%
$500–599	33%	36%
< $499	13%	15%
NA	0%	0%
*Number* *of* *cigarettes/day*		
< 15	53.8%	38%
15–25	34.6%	35%
26–35	9%	13%
36–40	2.6%	5%
N/A	0%	9%
*Total* *years* *tobacco* *smoking*		
< 10	31.3%	18%
10–20	11.3%	13%
21–30	28.7%	26%
31–40	18.7%	25%
41–50	7.5%	11%
51–60	2.5%	4%
N/A	0%	4%
*Food* *insecurity*		
Always (100% of the time)	18%	18%
Most of the time (75–99%)	10%	9%
Usually (50–75% of the time)	12%	13%
Sometimes (25–50% of the time)	25%	29%
Occasionally (< 25% of the time)	15%	13%
Never	18%	16%
NA	2%	2%
*Self-reported* *lung* disease*	21.9%	23.6%
*Can-COLD*		
Cough (without cold)	64%	62%
< 2 years	8%	9%
2–5 years	13%	13%
< 5 years	31%	29%
Phlegm (without cold)	70%	65%
< 2 years	17%	16%
2–5 years	9%	7%
< 5 years	20%	20%
Wheezing/whistling	72%	67%
Wheezing with cold	33%	33%
Shortness of Breath	39%	35%
Unable to Walk	36%	35%
*CAT* *Score* *(mean/SD)*	25.78 (8.45)	17.16 (8.11)
Cough	4.03 (1.36)	3.00 (1.39)
Phlegm	3.92 (1.46)	2.95 (1.52)
Chest	3.06 (1.62)	1.98 (1.57)
Walk	3.34 (1.85)	2.25 (1.86)
Activities	2.55 (1.60)	1.44 (1.50)
Confident	2.47 (1.83)	1.40 (1.74)
Sleep	3.25 (1.88)	2.07 (1.76)
Energy	3.21 (1.61)	2.11 (1.59)

*Asthma, *Chronic* *Obstructive* *Lung* *Disease,* *Emphysema* *and/or* *Lung* *Cancer*

There was no significant difference in R_5–20_, A_x_ and X_5_ values when comparing the oscillometry values amongst participants with and without a diagnosis of COPD (using fixed ratio (n = 32) and LLN (n = 24) (Table 2). Furthermore, in individuals diagnosed with COPD based on spirometry, there was no significant difference between the pre- and post- bronchodilator values of R_5–20_ and A_x_ (*p* = 0.63 and 0.43, respectively based on the fixed ratio method; and *p* = 0.45 and 0.36, respectively based on the LLN method), as would be expected.

**Table 2:** Mean R5-20, Ax and X5 values based on spirometry diagnosis of COPD

	Diagnosis of COPD using the fixed ratio method		Diagnosis of COPD using the LLN method	
	COPD	No COPD	COPD	No COPD
R_5–20_ *(cmH2O.s/L)*	1.02^a^	0.81^a^	1.20^b^	0.72^b^
A_x_ *(cmH2O/L)*	14.8^c^	13.0^c^	17.7^d^	11.2^d^
X_5_ *(cmH2O.s/L)*	− 1.43^e^	− 1.53^e^	− 1.47^f^	− 1.44^f^

*Mean* *oscillometry* *values* *are* *reported* *for* *R*5–20*,* *Ax* *and* *X*5 *based* *on* *the* *presence* *of* *COPD* *assessed* *using* *the* *fixed* *ratio* *method* *(a* *post-bronchodilator* *FEV1/FVC* *ratio* < 0.70*)* *and* *the* *LLN* *method* *(a* *post-bronchodilator* *FEV1/FVC* *ratio* ≤ *LLN).* *R5-20* *is* *a* *measure* *of* *small* *airway* *resistance.* *Ax* *is* *a* *measure* *of* *the* *area* *under* *the* *reactance* *curve.* *X5* *is* *a* *measure* *of* *airway* *elastance.* *P* *values* *were* *generated* *using* *a* *two-tailed* *t-test.* ^*a*^*p* = 0.48; ^*b*^*p* = 0.13; ^*c*^*p* = 0.70; ^*d*^*p* = 0.22; ^*e*^*p* = 0.77; ^*f*^*p* = 0.92. *n* = 55

**Fig. 1:**
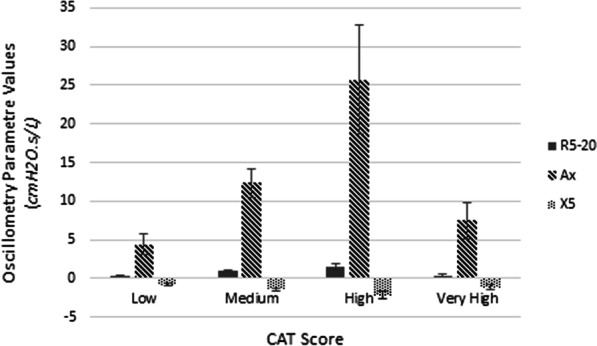
Mean R_5–20_, A_x_ and X_5_ values based on the COPD assessment test (CAT) score. Mean oscillometry values are reported for R_5–20_, A_x_ and X_5_ based on the CAT Score. R_5–20_ is a measure of small airway resistance. A_x_ is a measure of the area under the reactance curve. X_5_ is a measure of airway elastance. CAT score is classified as low (< 10), medium (10–20), high (21–30) and very high (> 30). *p* Values were generated using the ANOVA test. *p* Values for R_5–20_, A_x_ and X_5_ based on CAT score were 0.009, 0.007, and 0.05, respectively. Sample size based on CAT score group were as follows, low *n* = 10, medium *n* = 29, high *n* = 15, and very high *n* = 5

There was a significant difference in all three of the oscillometry parameters, R_5–20_, A_x_ and X_5_, based on CAT score (*p* = 0.009, 0.007 and 0.05, respectively) (Fig. 1). In a subgroup analysis, each parameter was compared to the presence or absence of individual symptoms. There was a significant difference in R_5–20_ and A_x_ based on the presence of phlegm (*p* = 0.03 and 0.02, respectively) and the presence of wheeze (*p* = 0.05 and 0.01, respectively) (Table 3). There was no significant difference in R_5–20_ or A_x_ based on the presence of cough or shortness of breath (Table 3). X_5_ did not correlate with any of the individual symptoms (Table 3). Lastly, there was no significant difference in oscillometry parameters amongst individuals diagnosed with asthma versus COPD.

**Table 3:** Mean R5-20, Ax and X*5* values based on individual symptoms

	Symptom							
	Cough	No cough	Phlegm	No phlegm	Wheeze	No wheeze	Shortness of breath	No shortness of breath
R5-20 *(cmH2O.s/L)*	1.14^a^	0.61^a^	**1.13**^**b**^	**0.54**^**b**^	**1.11**^**c**^	**0.58**^**c**^	1.41^d^	0.80^d^
Ax *(cmH2O/L)*	16.7^e^	9.04^e^	**17.0**^**f**^	**7.56**^**f**^	**17.1**^** g**^	**7.07**^** g**^	22.0^ h^	12.0^ h^
X*5* *(cmH2O.s/L)*	− 1.53^i^	− 1.29^i^	− 1.54^j^	− 1.13^j^	− 1.63^ k^	− 1.15^ k^	− 1.79^ l^	− 1.14^ l^

*Mean* *oscillometry* *values* *are* *reported* *for* *R*5–20*,* *Ax* *and* *X*5 *based* *on* *the* *presence* *of* *individual* *symptoms.* *R*5–20 *is* *a* *measure* *of* *small* *airway* *resistance.* *Ax* *is* *a* *measure* *of* *the* *area* *under* *the* *reactance* *curve.* *X*5 *is* *a* *measure* *of* *airway* *elastance.* *The* *presence* *of* *cough* *was* *defined* *as* *the* *presence* *of* *a* *cough* *on* *most* *days* *in* *the* *absence* *of* *a* *cold.* *The* *presence* *of* *phlegm* *was* *defined* *as* *the* *production* *of* *phlegm* *on* *most* *days* *in* *the* *absence* *of* *a* *cold.* *The* *presence* *of* *wheeze* *was* *defined* *as* *the* *presence* *of* *a* *wheeze* *any* *time* *in* *the* *last* 12 *months.* *The* *presence* *of* *shortness* *of* *breath* *was* *defined* *as* *activity* *limitation* *due* *to* *shortness* *of* *breath.* *P* *values* *were* *generated* *using* *a* *two-tailed* *t-test.* ^*a*^*p* = 0.06; ^*b*^*p* = 0.03; ^*c*^*p* = 0.05; ^*d*^*p* = 0.12; ^*e*^*p* = 0.07; ^*f*^*p* = 0.02; ^*g*^*p* = 0.01; ^*h*^*p* = 0.13; ^*i*^*p* = 0.42; ^*j*^*p* = 0.16; ^*k*^*p* = 0.15; ^*l*^*p* = 0.40. *n* = 56. *Bolded* *font* *indicates* *statistical* *significance*

## Discussion

To the best of our knowledge this is the first study employing oscillometry to assess lung disease burden in people who self-identified as homeless or at-risk for homelessness. A widening gap in socioeconomic status has resulted in a growing inequity in communities across North America. Shorter life expectancies, increased incidence of chronic diseases such as OLDs, higher rates of mental health issues, tobacco and substance use, and generally poorer quality of life are well documented in systematically disadvantaged urban populations who experience disadvantageous social determinants of health [28, 30, 40–42]. Previous data form the *Participatory* *Research* *in* *Ottawa:* *Management* *and* *Point-of-Care* *for* *Tobacco* *Dependence* (PROMPT) project demonstrated that the prevalence of disease burden, including cough, wheezing, phlegm, shortness of breath and COPD burden on everyday life (as measured by the CAT score), were two to three times greater in Ottawa’s systematically disadvantaged urban population than in the general Canadian population (based on the CanCOLD study). Despite greater disease burden, these urban populations are underrepresented in research leading to underdiagnosis and undertreatment of OLDs [28, 43].

Oscillometry has been shown to correlate with patient self-reported symptoms of COPD and asthma [9, 14, 15] and has been used to assess disease burden [16–19]. Compared to spirometry, oscillometry is easier to perform. Its ease of use makes it an attractive tool for assessing disease burden in various populations, including systematically disadvantaged populations, young children, and the elderly, and for monitoring disease burden in patients with chronic lung disease. Given the COVID-19 pandemic, there is an emerging need for minimizing aerosol generating testing, hence, oscillometry may be an alternative to spirometry as a safer test by minimizing infectious disease spread [13]. Furthermore, given that oscillometry is non-effort dependent, it may serve as a useful test for post-COVID-19 infected patients who have difficulty performing spirometry due to weakness. Limitations of oscillometry include, the paucity of data establishing diagnostic reference values, particularly in comorbid, non-Caucasian populations [27].

In our study, oscillometry values did not correlate with spirometry values, but were associated with the CAT score and individual symptoms of phlegm and wheeze, supporting previous data [14, 20] indicating that oscillometry is associated with symptom burden. Interestingly, A_x_, as an integrated measure of reactance, is more sensitive than X_5_ in discriminating between symptom groups. This is consistent with a number of other studies in asthma [44] and COPD patients [45]. Furthermore, previous studies have established a relationship between oscillometry parametres and small airway disease, suggesting that oscillometry may be able to detect changes in lung pathology prior to the onset of symptoms or changes in spirometry parametres [5]. The fact that oscillometry values were correlated with symptom burden, but not spirometry values, support the hypothesis that oscillometry may be better able to detect any respiratory disease compared to spirometry in an systematically disadvantaged urban population. The 6-min walk test (a well validated test for multiple diseases such as COPD, interstitial lung disease, pulmonary hypertension, recovery from prolonged hospitalization and mechanical ventilation) evaluates all the physiological systems involved in exercise tolerance (rather than providing specific information on each system or disease individually) [46]. Like the 6-min walk test, oscillometry appears to be an indicator of respiratory diseases, but it is unknown if it can differentiate between specific disease entities such as small airway diseases (i.e., OLDs like COPD and asthma), central airway diseases or neuromuscular diseases affecting lung function. Nonetheless, we do know that oscillometry is associated with symptom burden, making it a useful effort-independent test in the assessment and monitoring of multiple lung diseases [20, 21, 46].

Given the uncertainty regarding the ability of oscillometry to differentiate between COPD and asthma diagnoses, we compared oscillometry parameters amongst patients diagnosed with COPD versus asthma on spirometry. Albeit a small sample size, we did not find a significant difference in oscillometry parameters amongst patients diagnosed with asthma versus COPD based on spirometry. To the best of our knowledge, our study is the first to report oscillometry values in a diverse systematically disadvantaged, urban population with high degree of comorbid diseases, tobacco, and substance use.

## Limitations

The limitations of this study include a single study-site (Ottawa, Canada), small sample size, the absence of a healthy comparison group and high attrition rate. Of the eighty participants, only fifty-five had spirometry and oscillometry data at baseline. Regardless, this study is important given the paucity of literature involving this study population. A previous PROMPT publication demonstrated that the consumption of tobacco and other illicit substances significantly decreased over the 6-month study period [30]. It would have been interesting to study the effect of tobacco smoking on oscillometry values over time, however, only twenty-six participants underwent oscillometry testing at the 6-month follow-up, due to limited funding. Furthermore, comparing the oscillometry results of the study population to a group of healthy controls would have allowed for a more robust analysis of the effect of substance use and lung disease on oscillometry parameters. The absence of a healthy control group is a limitation of this study. The operational definition of 'asthma' and ‘COPD' used in this study was based on spirometry alone. We acknowledge that this approach has limitations, because spirometric findings of obstructive lung disease could also be due to other diseases like bronchiectasis [47, 48]. Nonetheless, this study is the first to use the oscillometry technique in a systematically disadvantaged urban population. It demonstrated that oscillometry is feasible to administer in this challenging setting and that oscillometry values correlate with respiratory disease burden. Future studies are warranted to generate more robust data regarding the use of oscillometry in this systematically disadvantaged population with disproportionate disease burden.

## Conclusion

Despite the small study size, our study demonstrates that administering oscillometry is feasible and the values correlate with respiratory disease burden in Ottawa’s systematically disadvantaged urban population. Given its ease of use, oscillometry is an attractive tool for assessing and monitoring symptom burden in subjects at risk for developing OLD.

## Data Availability

The data set that supports the findings of this study are not publicly available. Data are however available from the corresponding author upon reasonable request.
